# Protective Effect of Daily Physical Activity Against COVID-19 in a Young Adult Population on Reunion Island

**DOI:** 10.3390/medsci13010028

**Published:** 2025-03-12

**Authors:** Camille Cazeneuve, David Couret, Gregorie Lebeau, Wildriss Viranaicken, Marie-Eve Mathieu, Florian Chouchou

**Affiliations:** 1 Laboratoire d’IngéniéRIe de la Santé, du Sport et de l’Environnement (IRISSE, EA4075), UFR des Sciences de l’Homme et de l’Environnement, Université de La Réunion, 117 rue du General Ailleret, 97430 Le Tampon, La Réunion, France; 2 Diabète Athérothrombose Réunion Océan Indien (DéTROI), Inserm UMR 1188, Campus Santé de Terre Sainte, Université de La Réunion, 97410 Saint-Pierre, La Réunion, France; 3 School of Kinesiology and Physical Activity Sciences, Université de Montréal, Montréal, QC H3T 1J4, Canada; 4 Centre de recherche Azrieli, CHU Saint-Justine, Montréal, QC H3T 1C5, Canada

**Keywords:** physical activity, COVID-19, immunity, sedentary behavior, viral infection

## Abstract

The global fight against pandemics is a major public health issue. Epidemiological studies showed a reduced risk of the coronavirus disease 2019 (COVID-19) severity with the practice of regular physical activity (PA) in clinical populations. Here, we investigated the effect of PA against COVID-19 in a young general population. **Methods**: Two hundred ninety volunteers over 18 years old from Reunion Island responded to an online survey concerning sociodemographic, lifestyle and clinical information. Daily PA was studied using the International Physical Activity Questionnaire short version (IPAQ) and classified by overall score and intensities of PA. **Results**: Among 290 responders [179 women, median age = 27.5 years (interquartile range = 21.3 years)], 141 (48.6%) reported COVID-19 infection. Multivariate logistic analysis adjusted for age, sex, body mass index, chronic disease and alcohol consumption showed that the number of days per week of regular intense PA was independently associated with a low risk of COVID-19 infection [odds ratio (OR) 0.86; 95% confidence interval (CI) 0.24 to 0.99; *p* = 0.030], while regular moderate PA was not [OR 1.10; 95%CI 0.97 to 1.23; *p* = 0.137]. **Conclusions**: In a population of young adults, regular intense PA could offer a protective effect against COVID-19. Additional research is required to confirm this association in various viral infections and elucidate the fundamental mechanisms involved.

## 1. Introduction

The emergence of the coronavirus disease 2019 (COVID-19) pandemic highlighted an urgent need for preventive strategies against viral diseases, such as wearing masks, vaccination or social distancing [1]. In the broader context of global warming, which promotes the emergence of new viruses, the research of novel, preventive and effective strategies remains pertinent [2]. In this sense, some epidemiological studies showed that regular physical activity (PA) protects against severe COVID-19 [3,4,5,6]. In a large American population including patients from a medical group, Sallis and colleagues first highlighted physical inactivity as an independently high risk factor for hospitalization, admission to intensive care or death related to COVID-19 [5]. This work was recently replicated by Steenkamp and colleagues, showing that PA of moderate intensity is associated with a protective effect against severe COVID-19 in a large South African population including members of a medical plan engaged in a health promotion program [6]. More recently, Vanhelst et al. demonstrated that PA had a beneficial effect against COVID-19, as identified through serological testing [7].

These clinical and epidemiological results are consistent with specific biochemical models regarding virus progression within organs and the corresponding immunological response [8,9]. PA is associated with higher mitochondrial activity [10]: regular PA has been shown to improve mitochondrial quality and function [11] and immune function is highly dependent on mitochondrial respiration [12]. Hence, it can be hypothesized that the level of oxygen consumption may serve as an indicator of an individual’s enhanced ability to combat certain viral infections. As oxygen consumption and PA are closely linked, with more active individuals generally exhibiting higher oxygen consumption [13,14], regular daily PA could potentially enhance this protective effect against viruses.

Although these clinical and experimental studies allow us to establish a relationship between PA and the risk of COVID-19 severity, those studies were based on clinical populations [4,5,15,16] and recruited through the health system [5,6]. It remains uncertain whether PA could have a protective effect on the general population. Furthermore, it is crucial to determine which types of daily PA have a beneficial effect against viral infections such as COVID-19. Currently, there is limited knowledge regarding the parameters of PA practice such as intensity. Accordingly, the objective of this work was to investigate the effect of overall and specific intensities of PA on COVID-19 infection in a general population. We hypothesized that an individual who engages in regular moderate or vigorous intensity PA would have a reduced risk of infection and progression to severe COVID-19.

## 2. Materials and Methods

### 2.1. Study Sample

Volunteers were contacted through professional, academic and social networks. An online survey was proposed to the adult population (age ≥ 18 years) living on Reunion Island (France) from 5 April 2022 to 20 June 2022 using the Framaforms^®^ platform version 1.0.3 (Framasoft, Lyon, France). We had 291 respondents to our questionnaire, and one was excluded because he did not live on Reunion Island. Of these 290 subjects, 179 were women (61.7%). The median age of this sample was 27.5 years (interquartile range (IQR) = 21.3 years). This study was conducted in accordance with the rules of the French Data Protection Authority (National Commission for Information Technology and Civil Liberties—CNIL) and with the Declaration of Helsinki. In accordance with French law, we obtained an agreement from the delegated committee of the CNIL within our institution. All subjects gave their written informed consent to participate.

### 2.2. Online Survey

The online survey included questions on sociodemographics, lifestyle information, PA and clinical information such as COVID-19 infections and symptoms.

*Sociodemographic information*: Data on gender (male, female or other), date of birth, height, weight, qualifications based on French qualifications, i.e., without diploma; ‘certificat d’étude primaire’; ‘brevet des collèges/BEPC/DNB’; ‘CAP/BEP’ (or other technical diploma); ‘baccalaureat (BAC)’ (general, technological, professional); higher education diploma ‘BAC + 2; BAC + 3; BAC + 4; BAC + 5 or BAC + 7 et +)’, profession, annual household income, and postal code were collected via questionnaire.

*Lifestyle information*: Data on alcohol and cannabis consumption (never; occasionally; at least once a week; 2 or 3 times a week; every day or almost every day), smoking (nonsmoker; former smoker; smoker, even occasional or electronic cigarette) were collected. The PA was measured using the International Physical Activity Questionnaire (IPAQ) short version [17]. This questionnaire, composed of 7 questions, evaluates the level of PA and sedentary behaviors of an adult population over the past 7 days. This questionnaire is reliable and validated to define the quantity and intensity of PA, and is reproducible and can be administered to all subjects [17,18,19]. PA data were studied according to the overall and detailed scores. The short version of the IPAQ score proposes an overall score of daily PA being either low, moderate or high. Low corresponds to no PA reported or PA not reaching moderate or intense levels. Moderate corresponds to one of three criteria: (1) 3 or more days of intense physical activity lasting at least 20 min per day (min/d); (2) 5 or more days of moderate-intensity PA and/or walking lasting at least 30 min/d; (3) 5 or more days of PA combining walking and moderate- or high-intensity PA, reaching at least 600 metabolic equivalent of task (MET)-min/week. High corresponds to one of the two criteria: (1) intense physical activity at least 3 d/week, reaching at least 1500 MET-min/week; (2) 7 days of PA combining walking and moderate- or high-intensity PAs reaching at least 3000 MET-min. The detailed score provides information about the number of days and hours of moderate and intense PA per week.

*Clinical information*: Clinical data such as pregnancy, organ transplantation, chronic diseases (chronic obstructive pulmonary disease, cancer, metastatic cancer, renal disease, diabetes (1 or 2), hypertension, cardiovascular diseases) and chronic disease treatment were obtained via the questionnaire. On COVID-19 status: data about tests performed were obtained for each infection (antigen self-test; rapid antigen test; reverse transcriptase quantitative polymerase chain reaction (RT-qPCR); serological test; antigen self-test or RT-PCR; rapid antigen test + RT-PCR; antigen self-test + rapid antigen test + RT-PCR; or “I don’t know”). Symptoms of each infection (asymptomatic; fever; fatigue; cough; anosmia; ageusia; muscle aches; sore throat; headache; diarrhea; skin rash; discoloration of fingers and/or toes; red and/or irritated eyes; difficulties with breathing; chest pain; shortness of breath; loss of speech; functional impairment; confused state); symptom duration of each infection (1–3 days; 3–7 days; 1–2 weeks; 2–3 weeks; 3 weeks and more; or “I don’t know”); variant of each infection (alpha; beta; gamma; omicron; other; or “I don’t know”); hospitalization (yes or no or intensive care unit (ICU) admission); vaccination status (month, year and name of vaccine of each injection).

### 2.3. Statistical Analysis

The sample size was determined by a power analysis based on the published literature [20]. This analysis indicated that a sample size of 141 per group would provide over 90% power (with *p* < 0.05) to detect a difference in COVID-19 infection between active and inactive individuals.

Data were analyzed using Statview^®^ version 5.0.0.0 (SAS Institute, Inc., Cary, NC, USA). To assess the data’s normality, we used the Kolmogorov–Smirnov normality test. Sociodemographic, lifestyle and clinical data were compared according to COVID-19 status (COVID-19 infection report or not) using Pearson’s two-sided χ^2^ test for nominal data, and Mann–Whitney test analysis for continuous data. Continuous data were presented as median and IQR and nominal data as the number of subjects and percentages. The association between PA (overall and detailed scores) and COVID-19 was explored by univariate logistic analyses and multivariate logistic analyses, with a COVID-19 diagnosis as the dependent variable expressed as the odds ratio (OR) and using a 95% confidence interval (95%CI). To test the independence of the potential relationship between COVID-19 diagnosis and PA, age, gender, body mass index (BMI), chronic diseases, other diseases and alcohol consumption were included in the statistical model as independent variables [5]. All statistical tests were performed at *p* < 0.05.

## 3. Results

Two hundred and ninety-one volunteers answered the online survey and one subject was excluded (not living on Reunion Island). Out of all 290 participants, the majority were women (179 women, 61.7%), forming a group of median-age individuals (IQR 27.5–21.3 years old) with normal weight (BMI: 22.9 ± 4.8 kg/m^2^). A total of 36 subjects (12.4%) reported having chronic diseases and 141 (48.6%) reported past COVID-19 diagnosis. Statistical comparison according to COVID-19 status revealed no difference between gender (χ^2^ = 0.24, *p* = 0.624), chronic disease (χ^2^ = 2.58, *p* = 0.109), age (*p* = 0.419) or BMI (*p* = 0.611). Only alcohol consumption showed a significant statistical difference between the two groups; patients diagnosed with COVID-19 reported more frequent alcohol consumption (χ^2^ = 13.186, *p* = 0.010). Finally, for the IPAQ score, overall (χ^2^ = 1.058, *p* = 0.589) or detailed (the number of days of intense (*p* = 0.176) or moderate (*p* = 0.079) PA per week, the number of hours of intense (*p* = 0.502) or moderate (*p* = 0.083) PA per week) scores were not different according to COVID-19 status (Table 1).

Furthermore, univariate logistic analyses revealed no association between COVID-19 diagnosis and overall IPAQ score (*p* = 0.589). Similarly, these univariate analyses demonstrated no differences according to reported COVID-19 diagnosis: neither the number of days of intense (*p* = 0.164) or moderate (*p* = 0.110) PA per week, nor the number of hours of intense (*p* = 0.498) or moderate (*p* = 0.372) PA. Finally, multivariate logistic analyses controlled for the covariables revealed that the number of days of intense PA per week was associated with a decreased risk of COVID-19 diagnosis (OR 0.85, 95% CI: 0.74–0.98, *p* = 0.030), but not moderate PA (*p* = 0.137). The numbers of hours of intense (*p* = 0.126) and moderate (*p* = 0.630) PA per week were not associated with a reported COVID-19 diagnosis (Table 2).

## 4. Discussion

The present study investigated the effect of PA in a young adult population on Reunion Island against COVID-19. Based on previous epidemiological clinical data studied [3,4,5,6,7,15,16], we hypothesized that an individual who engages in regular moderate or intense PA would have a reduced risk of severe COVID-19. Here, we observed an independent association between COVID-19 diagnosis and intense regular PA, but not with moderate PA, as previously revealed in a clinical population. We can conclude that in the general population, protection through PA could be more pronounced when the activity is regular and intense than in a clinical population in which moderate intensity PA seems sufficient.

### 4.1. A Younger General Sample than Those in Clinical Studies

Since the onset of the COVID-19 pandemic, substantial research efforts have been undertaken to elucidate the mechanisms and behaviors capable of mitigating virus transmission, with particular focus on severe manifestations [21]. These studies have made it possible to demonstrate a protective effect of regular PA on mortality [5,6,22,23,24], admissions to ICU [5,6,22,23], hospitalizations [3,5,6,22,23], from reported diagnosis [3], or based on an RT-PCR [3,4,5,6,15,16] or serological test [3,7]. However, this understanding primarily relies on populations predominantly sourced from hospital settings, thereby complicating the assessment of the effect of PA on the general population that does not have access to hospitals [4,5,15]. In terms of public health, these studies showed that the World Health Organization’s recommendations for PA reduced the risk of COVID-19 severity [3,4,5,25,26]. In this context, our study, conducted on a sample of young individuals with a relatively low prevalence of chronic clinical conditions (12.4%), indicates that moderate-intensity PA may not provide sufficient protection, highlighting the potential necessity of engaging in intense PA to achieve COVID-19 protection. It is worth highlighting that the consistency of this vigorous activity is particularly significant, as it is only the frequency of intense PA per week that is associated with a decreased risk of being diagnosed with COVID-19. We did not observe any effect of gender or the presence of underlying comorbidities such as diabetes, hypertension or cardiovascular disease on COVID-19 infection, as can be seen in the literature. This may be explained by the fact that we are studying a young population and the proportion of these pathologies is low in our sample. However, we observed an effect of alcohol consumption on contracting COVID-19, potentially representing a more regular social and deleterious lifestyle to maximal oxygen consumption [3]. Consistently, an unfavorable lifestyle would lead to a higher risk of developing COVID-19.

PA enables individuals to develop higher maximal oxygen consumption than a sedentary lifestyle [13,14]. In young healthy individuals, maximum oxygen consumption increases considerably with intense PA [27], but moderate intensity exercise appears to be supportable in older subjects to improve their maximal oxygen consumption [28] and allows them to already benefit from a clinical gain [29]. In both cases, regular PA would improve mitochondrial qualities and functions [10], and it has been shown that immune function is highly dependent on mitochondrial respiration [12]. Thus, intense PA could be necessary for young subjects to improve mitochondrial respiration and protect them against infections, particularly those linked to SARS-CoV-2; whereas for an elderly population, moderate-intensity PA might be more relevant. These differences should undoubtedly be considered in future recommendations for PA and viral transmissions.

### 4.2. Interaction Between Mitochondrial Respiration and Immune Response

**Figure 1:**
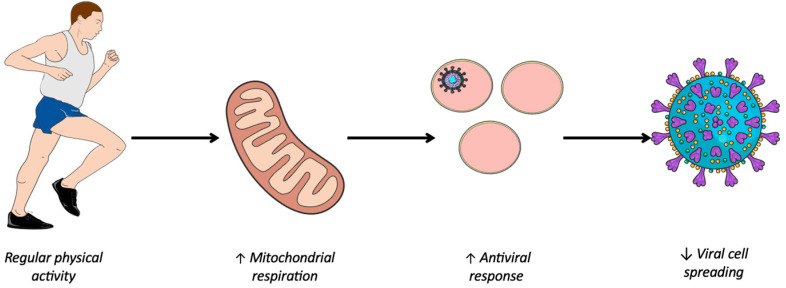
Improving immune system response through physical activity. ↑, *Improved*; ↓, *Reduced*.

Several viruses including SARS-CoV-2 have the ability to hijack the metabolism of infected cells to promote their replication [8,9]. Oxidative phosphorylation is the most important generator of ATP, producing 36ATP per molecule of glucose; whereas under anaerobic conditions, glycolysis produces just 2 ATP per molecule of glucose [30]. However, the latter favors the formation of biomass necessary for viral replication to the detriment of efficient energy production via oxidative phosphorylation [31]. This preference of the virus for aerobic glycolysis when the amount of oxygen is sufficient for oxidative phosphorylation is called the Warburg-like effect. First demonstrated in cancer cells [32], a phenomenon similar to the Warburg effect is suspected to be involved in virus-infected cells [33]. During viral infection, metabolic reprogramming towards aerobic glycolysis is one of the virus’ strategies for inhibiting the antiviral response. However, a study at the cellular level showed that enhanced oxidative phosphorylation was associated with an improved antiviral response [34,35]. This work is consistent with epidemiological studies showing a protective effect of PA [6,7] and that regular and moderate PA was associated with high mitochondrial activity, thus enhancing oxidative phosphorylation [10]. Hence, it seems that regular PA promotes mitochondrial respiration, which in turn enhances mitochondrial function, thereby contributing to the innate antiviral response (Figure 1).

### 4.3. Limitations and Perspectives

There are a number of limitations to this study. The limited size of our sample, which represents only 0.03% of the population of Reunion Island, and the small number of volunteers with chronic diseases restricted us from generalizing our results to the broader population and performing subgroup analyses (e.g., with and without chronic disease). However, our study sheds light on subjects who are typically underrepresented in clinical studies. PA is important and has to be considered for future recommendations in order to face future emerging viral diseases [2]: recent studies point to the fact that we are entering an era of pandemics originating from a cluster of events including climate disruption, increased international exchanges and urbanization [36]. Therefore, it seems necessary to implement a first-line strategy to which personalized PA can contribute in order to limit severe forms or the transmission of emerging infectious agents while awaiting effective antiviral or vaccine strategies.

## 5. Conclusions

This study showed that the number of days of intense PA is associated with a lower risk of COVID-19 diagnosis in a generally young population independently of known confounding factors (age, sex, BMI, chronic disease and alcohol consumption), but not moderate PA. Regular and intensive PA in a general population needs to be envisaged to deal with future emerging viral diseases in the context of global warming [36]. Further studies are required to better understand the place of PA in the fight against clinical warning and its viral consequences.

## Figures and Tables

**Table 1:** Demographic, clinical and physical activity data according to reported COVID-19 diagnosis.

	Groups	Total Research Sample (n = 290)	COVID-19 Diagnosis not Reported (n = 149)	COVID-19 Diagnosis Reported (n = 141)	*p* Value
Variables					
**Female**, *n (%)*		179 (61.7)	94 (32.4)	85 (29.3)	0.624
**Age** (y)		27.5 ± 21.3	27.8 ± 20.7	26.9 ± 22.3	0.419
**Body mass index** (kg/m^2^)		22.9 ± 4.8	23.0 ± 4.6	22.8 ± 5.0	0.611
**Alcohol** **Consumption**	Never, *n (%)*	71 (24.5)	46 (15.9)	25 (8.6)	**<0.05**
	Occasionally, *n (%)*	136 (46.9)	70 (24.1)	66 (22.8)	
	1 time/week, *n (%)*	48 (16.6)	19 (6.6)	29 (10.0)	
	2–3 times/week, *n (%)*	24 (8.3)	7 (2.4)	17 (5.9)	
	Every day or almost, *n (%)*	11 (3.8)	7 (2.4)	4 (1.4)	
**Chronic disease**, *n (%)*		36 (12.4)	23 (7.9)	13 (4.5)	0.109
**Chronic obstructive pulmonary disease**, *n (%)*		1 (0.3)	1 (0.3)	0 (0)	0.330
**Cancer**, *n (%)*		1 (0.3)	0 (0)	1 (0.3)	0.303
**Metastatic cancer**, *n (%)*		1 (0.3)	0 (0)	1 (0.3)	0.303
**Renal Disease**, *n (%)*		4 (1.4)	2 (0.7)	2 (0.7)	0.956
**Diabetes**, *n (%)*		1 (0.3)	1 (0.3)	0 (0)	0.330
**Hypertension**, *n (%)*		10 (3.4)	5 (1.7)	5 (1.7)	0.929
**Cardiovascular Disease**, *n (%)*		5 (1.7)	1 (0.3)	4 (1.4)	0.157
**Other Disease**, *n (%)*		32 (11.0)	20 (6.9)	12 (4.1)	0.182
**Overall** **IPAQ Score**	Low, *n (%)*	33 (11.4)	18 (6.2)	15 (5.2)	0.589
	Moderate, *n (%)*	89 (30.7)	49 (16.9)	40 (13.8)	
	High, *n (%)*	168 (57.9)	82 (28.3)	86 (29.6)	
**Detailed** **IPAQ Score**	Number of day(s) of intense PA /week	2.0 ± 3.0	2.0 ± 3.3	1.0 ± 3.0	0.176
	Number of hour(s) of intense PA /week	1.5 ± 4.5	1.5 ± 4.5	1.5 ± 4.5	0.502
	Number of day(s) of moderate PA /week	3.0 ± 2.0	2.0 ± 3.0	3.0 ± 2.0	0.079
	Number of hour(s) of moderate PA /week	2.5 ± 3.0	2.5 ± 2.0	2.5 ± 3.0	0.083

Continuous data are presented as median ± interquartile range and nominal data as number of subjects (percentages). Statistical differences were determined by Pearson’s two-sided χ^2^ test for nominal data and by Mann–Whitney test for continuous data. Abbreviations: PA, physical activity; IPAQ, International Physical Activity Questionnaire.

**Table 2:** Univariate and multivariate logistic analyses to explain reported COVID-19 diagnosis.

	Unadjusted OR (95% CI)	*p*	Adjusted OR (95% CI)	*p*
**Overall IPAQ Score**				
**Low**	1	-	1	-
**Moderate**	0.98 (0.44–2.19)	0.960	0.97 (0.42–2.25)	0.946
**High**	1.26 (0.60–2.66)	0.547	1.06 (0.48–2.37)	0.884
**Detailed IPAQ Score**				
**Intense PA**				
**Number of day(s) of intense PA/week**	0.92 (0.81–1.04)	0.164	**0.85 (0.74–0.98)**	**0.030**
**Number of hour(s) of intense PA/week**	0.98 (0.91–1.05)	0.498	0.94 (0.86–1.02)	0.126
**Moderate PA**				
**Number of day(s) of moderate PA/week**	1.10 (0.98–1.22)	0.110	1.10 (0.97–1.23)	0.137
**Number of hour(s) of moderate PA/week**	1.03 (0.95–1.10)	0.372	1.02 (0.95–1.10)	0.630

Adjusted multivariate logistic analyses for cofactors including age, sex, body mass index, chronic diseases, and alcohol consumption to explain reported COVID-19 diagnosis. Abbreviations: OR, odd ratio; CI, confidence interval; PA, physical activity; IPAQ, International Physical Activity Questionnaire.

## Data Availability

The anonymized data that support the findings of this study are available to qualified researchers upon reasonable request after approval by the corresponding author.

## Abbreviations

The following abbreviations are used in this manuscript:

COVID-19	Coronavirus disease 2019
PA	Physical activity
OR	Odds ratio
BAC	Baccalaureat
IPAQ	International Physical Activity Questionnaire
MET	Metabolic equivalent of task
RT-qPCR	Reverse transcriptase quantitative polymerase chain reaction
ICU	Intensive care unit
IQR	Interquartile difference
95%CI	95% confidence interval
BMI	Body mass index
